# Comorbid insomnia and sleep apnea (COMISA) and incident cardiovascular risk: a prospective analysis from the HypnoLaus cohort

**DOI:** 10.1016/j.eclinm.2026.104227

**Published:** 2026-09-28

**Authors:** Geoffroy Solelhac, Théo Imler, Nicola-Andrea Marchi, Sébastien Baillieul, Sandra Van Den Broecke, Gabriela Caetano, Grégory Heiniger, Adrien Waeber, Isabel Ericson, Virginie Bayon, Anne-Sophie Lombardi, José Haba-Rubio, Peter Vollenweider, Pedro Marques-Vidal, Raphaël Heinzer

**Affiliations:** a Center for Investigation and Research in Sleep, Lausanne University Hospital and University of Lausanne, Lausanne, Switzerland; b Grenoble Alpes University, HP2 Laboratory, INSERM U1300 and Grenoble Alpes University Hospital, Grenoble, France; c Department of Medicine, Internal Medicine, Lausanne University Hospital and University of Lausanne, Lausanne, Switzerland

**Keywords:** COMISA, Sleep, OSA, Sleep apnea, Insomnia, Cardiovascular risk

## Abstract

**Background:**

Comorbid insomnia and obstructive sleep apnea (COMISA) is a prevalent and clinically relevant phenotype. While both insomnia and OSA have been individually associated with increased cardiovascular risk, few longitudinal studies have evaluated their combined impact on incident cardiovascular events in the general population.

**Methods:**

A total of 1860 participants from the CoLaus|PsyCoLaus cohort, originally recruited between 2003 and 2008 and assessed in the HypnoLaus study between 2009 and 2013, were included, all of whom underwent full-night polysomnography and completed the Pittsburgh Sleep Quality Index (PSQI). OSA was defined as an apnea-hypopnea index (AHI) ≥15 events/h. Insomnia symptoms were defined as difficulty initiating or maintaining sleep ≥3 times/week over the past month. Participants were classified into four groups: reference (no insomnia, no OSA), insomnia alone, OSA alone, and COMISA. Incident cardiovascular events (coronary heart disease, myocardial infarction, stroke, or cardiovascular death) were adjudicated by an expert committee over a median follow-up period of 7.92 years [IQR: 6.30–8.77]. Cox regression models were adjusted for demographic, behavioral, and clinical covariates.

**Findings:**

Compared to the reference group, COMISA was independently associated with an increased risk of incident cardiovascular events (HR 1.78, 95% CI: 1.09–2.92, p = 0.021), while no significant association was observed for insomnia (HR 0.59, 95% CI: 0.31–1.11, p = 0.10) or OSA alone (HR 1.33, 95% CI: 0.84–2.10, p = 0.22). A significant interaction between OSA and insomnia was observed (p = 0.035). Results were consistent in participants without prior cardiovascular events and across alternative definitions of COMISA.

**Interpretation:**

COMISA, but not OSA or insomnia alone, was associated with increased incident cardiovascular events. These findings highlight the need to identify and treat COMISA as a distinct and high-risk phenotype in cardiovascular prevention strategies.

**Funding:**

Faculty of Biology and Medicine of Lausanne, Lausanne University Hospital, Swiss National Science Foundation, Leenaards Foundation, GlaxoSmithKline, Ligue Pulmonaire Vaudoise.

**Research in context**

**Evidence before this study**

We initially searched PubMed on May 1, 2024 and updated the search on October 31, 2025, without language restrictions, using the terms “(comorbid insomnia and sleep apnea OR COMISA OR insomnia AND obstructive sleep apnea) AND (cardiovascular disease OR cardiovascular events OR myocardial infarction OR coronary heart disease OR stroke OR hypertension OR cardiovascular mortality)”. We also screened reference lists of relevant articles. We included longitudinal studies assessing cardiovascular outcomes in adults with COMISA. Four studies were identified. Overall, COMISA was associated with increased cardiovascular risk, but the evidence was heterogeneous regarding populations and definitions of insomnia and OSA. Only two studies objectively assessed OSA using polysomnography, while others relied on diagnostic codes or electronic health records. Associations were not consistently maintained after adjustment for cardiovascular risk factors, and some studies did not separately assess insomnia and OSA alone. Given this heterogeneity, a pooled estimate was not considered appropriate.

**Added value of this study**

In a population-based cohort with full-night polysomnography and adjudicated cardiovascular events, COMISA, but neither OSA nor insomnia symptoms alone, was independently associated with incident cardiovascular events over a median follow-up of 7.92 years. This association was robust across alternative insomnia definitions and among participants without previous cardiovascular events.

**Implications of all the available evidence**

Together with previous studies, our findings support COMISA as a clinically relevant phenotype associated with increased cardiovascular risk. Systematic consideration of insomnia symptoms in OSA, and of OSA in insomnia, may improve risk stratification. Further studies should determine whether combined treatment of both disorders can reduce cardiovascular risk.

## Introduction

Comorbid insomnia and obstructive sleep apnea (COMISA) is a frequent and clinically significant sleep disorder phenotype, defined by the coexistence of insomnia symptoms and obstructive sleep apnea (OSA).^1^ Both conditions are highly prevalent in the general population and have independently been associated with increased cardiovascular risk.^2^, ^3^, ^4^, ^5^ Emerging evidence suggests that their co-occurrence may confer a greater health burden than either condition alone, potentially due to additive or synergistic pathophysiological mechanisms.^6^^,^^7^ From a pathophysiological perspective, insomnia-related hyperarousal and sleep fragmentation may interact with OSA-related intermittent hypoxemia and intrathoracic pressure changes, leading to sympathetic activation, inflammation, and hypothalamic-pituitary-adrenal axis dysregulation, which may contribute to increased cardiovascular risk.^8^^,^^9^

Recent studies have shown that up to 30–50% of individuals with OSA report significant insomnia symptoms, and vice versa, highlighting a substantial overlap between the two conditions.^10^ Despite this high prevalence, COMISA remains underdiagnosed and inconsistently defined in research settings, particularly in population-based studies where reliance on self-reported measures and varying OSA diagnostic thresholds limits comparability.

In a previous cross-sectional analysis of the HypnoLaus cohort, COMISA was found to be associated with an increased prevalence of hypertension and metabolic syndrome.^6^ While such cross-sectional findings provide important insights into potential associations between COMISA and cardiovascular risk factors, longitudinal data are needed to establish temporality and explore potential causal mechanisms. To date, only a limited number of prospective studies have examined whether COMISA independently predicts incident cardiovascular events.^11^, ^12^, ^13^, ^14^

The present study aimed to investigate the longitudinal association between COMISA and the incidence of cardiovascular events in the population-based HypnoLaus cohort over a median follow-up period of 7.92 years [IQR: 6.30–8.77].

We hypothesized that COMISA would be associated with an increased risk of incident cardiovascular events compared with the reference group.

## Methods

### Participants

The CoLaus|PsyCoLaus cohort study was designed to assess associations between mental disorders and cardiovascular risk factors in the community.^15^ Participants were randomly selected from 35- to 75-year-old residents of Lausanne, Switzerland, between 2003 and 2008, using the official population registry to ensure a representative sample of the general population. They underwent thorough physical and psychiatric evaluations at baseline and at three follow-up visits, conducted approximately every 5 years (first follow-up: 2009–2013, second follow-up: 2014–2018, third follow-up: 2018–2022). The sleep investigations (polysomnography, PSG) within the HypnoLaus nested study took place between 2009 and 2013, i.e., between the first and second follow-up of the CoLaus|PsyCoLaus study.^16^ This analysis included participants who underwent a PSG investigation and completed the Pittsburgh Sleep Quality Index (PSQI)^17^ during the first follow-up of CoLaus|PsyCoLaus. Questionnaires, including the PSQI, were administered in French.

Prospective data on cardiovascular events were obtained from the third follow-up (2018–2022) of the CoLaus|PsyCoLaus study. The institutional Ethics Committee of the University of Lausanne, subsequently the Ethics Commission of Canton Vaud, approved the CoLaus|PsyCoLaus study, including its first (reference 33/09) and third (reference PB_2018-00040) follow-ups; approval for the overall study was confirmed in 2021 (project PB_2018-00038, reference 239/09). The study was conducted in accordance with the Declaration of Helsinki and applicable Swiss legislation. All participants provided written informed consent.

The primary analytic sample size was determined by the number of participants with available polysomnography, cardiovascular outcome data, and sufficient PSQI information for insomnia classification. Participants with insufficient information to classify insomnia status were excluded. Participants with a polysomnography-derived total sleep time <240 min were excluded from the primary analysis but included in a sensitivity analysis.

### OSA assessment

Participants underwent a full-night PSG. PSG data were visually scored according to the American Academy of Sleep Medicine (AASM) guidelines (2007).^18^ Apneas and hypopneas were defined according to the 2012 AASM manual to calculate an apnea-hypopnea index (AHI = number of respiratory events per hour).^19^ OSA was defined with an AHI ≥15/events/h of sleep.

### Insomnia assessment

In the cohort, insomnia symptoms were assessed using the PSQI,^17^ based on participants’ responses to questions about sleep initiation and sleep maintenance/early morning awakenings.

Insomnia symptoms were defined according to PSQI questions 5a and 5b, defined below. Participants were classified as having insomnia symptoms if they reported experiencing at least one of the following symptoms ≥3 times per week during the past month:

• Difficulty falling asleep within 30 min (PSQI 5a)
• Waking up in the middle of the night or early morning (PSQI 5b)

Alternative insomnia definitions were considered for sensitivity analyses.

Insomnia including sleep medication use: Participants were considered as having insomnia symptoms if they met the primary criteria and/or reported using sleep medication ≥3 times per week during the past month, based on PSQI question 7.

Insomnia including prolonged wake after sleep onset (WASO): Participants were considered to have insomnia symptoms if they met the primary criteria and reported a WASO ≥30 min. WASO was estimated using PSQI questions one to four (bedtime, wake time, sleep latency, and sleep duration) to avoid the potential limitations of PSQI question 5b, which may capture brief awakenings related to obstructive sleep apnea rather than clinically meaningful insomnia complaints. A subgroup with insomnia symptoms was also defined based on the same criteria, combined with an objectively measured WASO ≥30 min according to PSG.

### Definition of study groups

Participants were classified into four groups based on the presence of insomnia symptoms and OSA:

1. Reference: No insomnia symptoms and no OSA (AHI <15/events/h)
2. Insomnia: Insomnia symptoms and no OSA (AHI <15/events/h)
3. OSA: OSA (AHI ≥15/events/h) and no insomnia symptoms
4. COMISA: Co-occurrence of insomnia symptoms and OSA (AHI ≥15/events/h)

The same approach was used to define COMISA when using alternative insomnia definitions. Throughout the manuscript, the term insomnia refers to insomnia symptoms.

### Cardiovascular risk assessment and relevant covariates

The following covariates were selected based on their established association with cardiovascular risk: age, sex, body mass index (BMI), weekly alcohol consumption, smoking status, previous history of cardiovascular event, hypertension, diabetes and dyslipidemia. Lifestyle-related factors including smoking, alcohol intake, and BMI were assessed using a standardized interview and physical examination. BMI was categorized as normal (<25 kg/m^2^), overweight (≥25 to <30 kg/m^2^) and obese (≥30 kg/m^2^). Smoking status was defined as current versus non-current smoking. Hypertension was defined as either a systolic blood pressure ≥140 mm Hg, a diastolic blood pressure ≥90 mm Hg, or the current use of antihypertensive drug treatment. Diabetes was defined as either a fasting blood glucose ≥7 mmol/L or the current use of antidiabetic drug treatment. Dyslipidemia was defined as the presence of any of the following: high LDL-cholesterol (≥4.1 mmol/L) or low HDL-cholesterol (<1.0 mmol/L), high triglyceride levels (≥2.2 mmol/L), or the current use of hypolipidemic drug treatment. Previous history of cardiovascular events was defined using the same criteria as the cardiovascular outcome, including myocardial infarction, stroke (ischemic, hemorrhagic, or central retinal artery occlusion), and coronary heart disease.

### Cardiovascular events outcome

The composite incident cardiovascular endpoint encompassed both fatal and nonfatal cardiovascular events (Supplementary Table S1). Fatal events included cardiovascular deaths due to myocardial infarction or stroke, while nonfatal events comprised myocardial infarction, stroke, and coronary heart disease. Strokes included ischemic and hemorrhagic strokes, as well as central retinal artery occlusion.

Cardiovascular events were identified through a standardized multi-step procedure combining participant reports, systematic review of medical records, hospital database searches using ICD-10 codes, and mortality registries. All relevant documentation (clinical records, laboratory data, imaging, ECG, and procedural reports) was collected when necessary. Events were adjudicated by a local expert committee composed of internists, cardiologists, and neurologists, in accordance with international guidelines.^20^ Coronary events were reviewed by cardiologists, stroke events by a neurologist, and deaths by internists using all available information. Myocardial infarction was defined according to the universal definition, requiring a rise and/or fall in cardiac troponins above the 99th percentile together with evidence of myocardial ischemia, or based on clinical, ECG, and imaging criteria when biomarkers were unavailable.

The composite variable was constructed based on all available validated events, with only the first event per participant considered in the analysis.^21^

### Statistical analysis

Baseline characteristics of participants in the four groups (reference, insomnia, OSA, and COMISA) were compared using Kruskal–Wallis for continuous variables and chi-squared tests for categorical variables. For descriptive variables with missing data, percentages were calculated using the number of participants with available data as the denominator and presented as n/N (%). Pairwise comparisons were corrected for multiple comparisons using Benjamini-Hochberg method.

As an initial analysis, the association between moderate-to-severe OSA (AHI ≥15 events/h), irrespective of insomnia symptoms, and incident cardiovascular events was assessed using a fully adjusted Cox proportional hazards model. Participants were classified into four groups (reference, insomnia, OSA, and COMISA). Kaplan–Meier survival curves were used to illustrate time-to-event data across the four groups, and differences were assessed using the log-rank test.

Sleep disorder group (reference, insomnia alone, OSA alone, and COMISA) was included as a categorical predictor in Cox proportional hazards models, with participants without insomnia and without OSA as the reference category. Unadjusted Cox proportional hazards models were also performed for the primary exposure. Additional analyses were conducted using alternative definitions of insomnia, including the incorporation of sleep medication use, subjective prolonged wake after sleep onset (WASO), and objective prolonged WASO.

Models were adjusted for age (continuous), sex (binary), body mass index (categorical), diabetes (binary), dyslipidemia (binary), hypertension (binary), smoking status (binary), previous history of cardiovascular events (binary), and alcohol intake (continuous). The proportional hazards assumption was assessed using a Schoenfeld’s test. Linearity of continuous covariates was assessed using Martingale residual plots and visual inspection of functional form plots. Results are expressed as hazard ratios (HRs) with 95% confidence intervals (95% CIs). Adjusted absolute risks of cardiovascular events at 8 years were estimated from the fully adjusted Cox model by averaging predicted risks across the analytic sample.

To assess the interaction between OSA and insomnia, a Cox proportional hazards model included binary indicators for OSA and insomnia and an OSA × insomnia interaction term. OSA was coded as present in participants with OSA alone or COMISA, and insomnia as present in participants with insomnia alone or COMISA. The interaction term was positive only when both conditions were present and represents the non-additive effect of OSA and insomnia in the proportional hazards model, with its hazard ratio quantifying the additional change in hazard beyond their individual effects. The interaction term was tested in the fully adjusted model, and its statistical significance was evaluated using the corresponding p-value.

Descriptive analyses, Kaplan–Meier curves and unadjusted Cox proportional hazards models included all 1860 participants. Adjusted models were performed using complete cases for the covariates included in each model, and the corresponding analytic sample size is reported for each analysis. Analyses using alternative insomnia definitions were restricted to participants with sufficient information to apply all three definitions. The number and proportion of missing values for each variable are reported in Supplementary Table S2. As a secondary analysis, models were repeated in participants free of prior cardiovascular events at baseline.

Sensitivity analyses were conducted after excluding participants who initiated CPAP treatment during follow-up, without excluding participants with total sleep time <240 min, and by restricting the outcome to cardiac events only (i.e., excluding cerebrovascular events), both in the full sample and in the subgroup without prior cardiovascular events. A separate Cox proportional hazards model for cerebrovascular events was not performed due to the limited number of events.

All analyses were performed using R software version 4.3.0 (R Foundation, Vienna, Austria).^22^

### Role of the funding source

The Faculty of Biology and Medicine of Lausanne, the Lausanne University Hospital (CHUV), Leenaards Foundation, and Ligue Pulmonaire Vaudoise funded the salary of the technicians who did the sleep recordings. The Swiss National Science Foundation funded the statisticians and supported the initial CoLaus cohort. GlaxoSmithKline supported the initial CoLaus cohort and funded the polysomnography recorders. The funders of the study had no role in study design, data collection, data analysis, data interpretation, or writing of the report. The corresponding author had full access to all data in the study and had final responsibility for the decision to submit for publication.

## Results

### Study population

A total of 1860 participants (Fig. 1) were included in the analysis, with a median age of 56.5 years (interquartile range (IQR): 48.6–66.3), 980 (52.7%) of them were women, the median follow-up duration was 7.92 years [IQR: 6.30–8.77]. In this population, 606 participants (32.6%) had insomnia and 650 participants (34.9%) had moderate-to-severe OSA (AHI ≥15 events/h). Among those with OSA, the median AHI was 25.9 events/h [19.2–38.0], with a median hypoxic burden of 33.4%.min/h [20.3–54.1] and median T90 of 1.80% [0.38–7.3], indicating a significant level of respiratory disturbance and nocturnal hypoxemia. Baseline characteristics of all participants and of the four groups (reference, insomnia, OSA, and COMISA) are presented in Table 1.

**Fig. 1:**
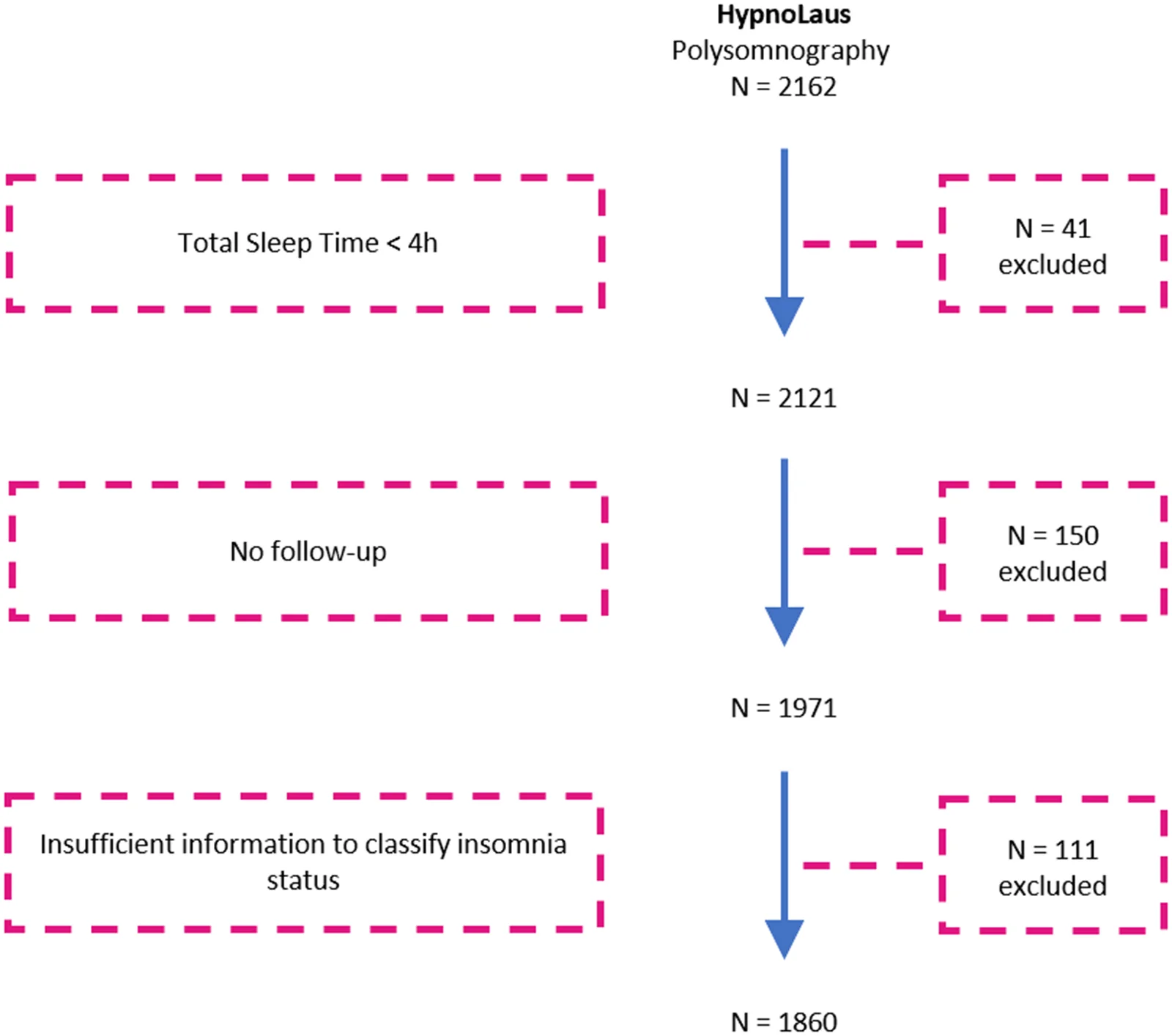
**Flowchart of the study**.

**Table 1:** Baseline characteristics of participants according to sleep group (reference, insomnia, OSA, and COMISA).

	All N = 1860	R: Reference N = 815 (43.8%)	I: Insomnia N = 395 (21.2%)	O: OSA N = 439 (23.6%)	C: COMISA N = 211 (11.3%)	p
Age (years, median [Q1–Q3])	56.5 [48.6–66.3]	52.8 [46.3–63.3]^C,I,O^	55.6 [48.0–63.9]^R,C,O^	61.4 [53.4–68.4]^R,I^	63.7 [55.8–69.2]^R,I^	<0.0001
Women, n/N (%)	980/1860 (52.7%)	487/815 (59.8%)^C,I,O^	273/395 (69.1%)^R,C,O^	145/439 (33.0%)^R,I^	75/211 (35.5%)^R,I^	<0.0001
BMI categories, n/N (%)						<0.0001
Normal, <25 kg/m^2^	818/1849 (44.2%)	470/813 (57.8%)	200/393 (50.9%)	94/434 (21.7%)	54/209 (25.8%)	
Overweight, ≥25 and <30 kg/m^2^	744/1849 (40.2%)	271/813 (33.3%)	143/393 (36.4%)	224/434 (51.6%)	106/209 (50.7%)	
Obese, ≥30 kg/m^2^	287/1849 (15.5%)	72/813 (8.86%)	50/393 (12.7%)	116/434 (26.7%)	49/209 (23.4%)	
Current smokers, n/N (%)	331/1860 (17.8%)	159/815 (19.5%)	64/395 (16.2%)	75/439 (17.1%)	33/211 (15.6%)	0.37
Alcohol consumption (unit/week), median [Q1–Q3]	4.00 [1.00–9.00]	4.00 [1.00–8.00]^C,O^	4.00 [1.00–8.00]^C,O^	5.00 [2.00–10.0]^R,I^	6.00 [2.00–11.0]^R,I^	0.0001
Diabetes, n/N (%)	170/1858 (9.2%)	41/815 (5.0%)^C,O^	26/395 (6.6%)^C,O^	70/437 (16.0%)^R,I^	33/211 (15.6%)^R,I^	<0.0001
Hypertension, n/N (%)	735/1858 (39.6%)	246/814 (30.2%)^C,O^	123/395 (31.1%)^C,O^	242/438 (55.3%)^R,I^	124/211 (58.8%)^R,I^	<0.0001
Dyslipidemia, n/N (%)	479/1858 (25.8%)	167/815 (20.5%)^C,O^	102/395 (25.8%)^O^	148/437 (33.9%)^R,I^	62/211 (29.4%)^R^	<0.0001
Previous history of CVE, n/N (%)	55/1860 (3.0%)	10/815 (1.2%)^C,O^	5/395 (1.3%)^C,O^	25/439 (5.7%)^R,I^	15/211 (7.1%)^R,I^	<0.0001
AHI (events/h), median [Q1–Q3]	9.60 [4.00–20.1]	5.80 [2.75–9.50]^C,O^	5.40 [2.40–9.20]^C,O^	25.5 [19.0–37.7]^R,I^	26.7 [19.6–38.7]^R,I^	<0.0001
ODI 3% (events/h), median [Q1–Q3]	9.60 [4.20–18.8]	5.60 [2.80–9.40]^C,O^	5.40 [2.70–9.20]^C,O^	23.1 [17.6–35.4]^R,I^	25.4 [17.9–35.3]^R,I^	<0.0001
ODI 6% (events/h), median [Q1–Q3]	0.60 [0.00–2.52]	0.20 [0.00–0.60]^C,O^	0.10 [0.00–0.60]^C,O^	4.30 [1.75–9.00]^R,I^	4.30 [1.80–10.2]^R,I^	<0.0001
PLMSI (events/h), median [Q1–Q3]	2.33 [0.00–19.1]	1.69 [0.00–16.7]^C,O^	1.94 [0.00–13.9]^C,O^	3.83 [0.00–24.1]^R,I^	4.51 [0.00–27.4]^R,I^	0.0012
Respiratory arousal index (events/h), median [Q1–Q3]	4.30 [1.48–9.80]	2.30 [0.90–4.50]^C,O^	2.00 [0.70–4.00]^C,O^	13.1 [8.70–19.9]^R,I^	14.2 [9.65–21.7]^R,I^	<0.0001
Mean SpO_2_ (%), median [Q1–Q3]	94.3 [93.2–95.3]	94.9 [93.9–95.7]^C,O^	94.6 [93.7–95.7]^C,O^	93.5 [92.4–94.4]^R,I^	93.4 [92.4–94.3]^R,I^	<0.0001
Hypoxic burden (%.min/h), median [Q1–Q3]	10.0 [3.17–24.8]	5.48 [1.85–11.7]^C,O^	4.67 [1.83–11.0]^C,O^	32.7 [20.2–53.5]^R,I^	34.8 [21.1–56.0]^R,I^	<0.0001
PWAD (events/h), median [Q1–Q3]	51.5 [37.6–65.4]	51.0 [37.2–63.9]	53.2 [37.5–66.3]	51.6 [39.1–66.1]	51.6 [37.1–69.6]	0.47
ΔHR (events/h), median [Q1–Q3]	10.9 [8.62–13.7]	10.9 [8.46–13.6]	9.91 [8.28–12.4]^C,O^	11.5 [8.99–14.4]^I^	11.0 [8.99–14.2]^I^	0.0024
T90 (%), median [Q1–Q3]	0.10 [0.00–1.70]	0.00 [0.00–0.20]^C,O^	0.00 [0.00–0.40]^C,O^	1.80 [0.40–6.60]^R,I^	2.10 [0.30–8.25]^R,I^	<0.0001
Baseline heart rate (BPM), median [Q1–Q3]	63.1 [57.5–68.3]	63.1 [57.1–68.2]	63.1 [58.4–68.4]	62.9 [58.0–68.9]	63.1 [57.2–67.5]	0.82
RMSSD (ms), median [Q1–Q3]	27.8 [20.4–39.3]	28.2 [21.3–41.0]	27.5 [19.9–38.4]	28.2 [20.3–38.1]	24.9 [19.6–36.3]	0.059
SDNN (ms), median [Q1–Q3]	79.2 [64.5–96.7]	78.5 [65.3–97.3]^I^	75.8 [60.9–93.1]^R,C,O^	82.0 [66.8–97.5]^I^	80.2 [65.0–98.1]^I^	0.0085
pNN50 (%), median [Q1–Q3]	6.11 [2.11–15.2]	6.50 [2.40–16.7]	6.05 [1.74–14.3]	6.09 [2.21–14.5]	4.31 [1.80–11.8]	0.031
TST (min), median [Q1–Q3]	406 [362–447]	412 [366–457]^C,O^	407 [369–450]^C,O^	398 [351–440]^R,I^	392 [350–437]^R,I^	0.00017
N1 (%), median [Q1–Q3]	10.2 [7.29–14.7]	9.20 [6.71–12.7]^C,O^	9.10 [6.62–12.9]^C,O^	13.2 [8.96–18.7]^R,I^	13.4 [9.10–17.8]^R,I^	<0.0001
N2 (%), median [Q1–Q3]	45.7 [40.0–52.3]	45.1 [40.0–51.2]^C^	45.9 [39.6–51.1]^C,O^	45.9 [39.6–54.1]^I^	48.5 [41.2–56.6]^R,I^	0.0016
N3 (%), median [Q1–Q3]	19.3 [14.1–24.9]	20.6 [15.8–25.8]^C,O^	20.4 [16.1–26.0]^C,O^	16.8 [11.9–22.3]^R,C,I^	14.7 [10.6–20.5]^R,I,O^	<0.0001
REM (%), median [Q1–Q3]	22.4 [18.4–26.0]	23.0 [19.5–26.4]^C,O^	23.5 [19.4–26.5]^C,O^	20.8 [16.1–25.2]^R,I^	20.2 [15.9–24.6]^R,I^	<0.0001
Sleep latency (min), median [Q1–Q3]	10.9 [4.60–20.7]	10.1 [4.50–19.9]	11.9 [5.45–21.2]	10.4 [3.90–20.4]	12.1 [5.35–27.1]	0.041
WASO (min), median [Q1–Q3]	56.0 [33.3–95.6]	45.6 [28.9–78.6]^C,I,O^	57.0 [33.4–92.0]^R,C,O^	69.5 [39.8–108]^R,C,I^	77.0 [46.0–119]^R,I,O^	<0.0001
REM latency (min), median [Q1–Q3]	75.5 [59.5–108]	73.5 [59.0–103]	74.0 [59.5–102]	81.0 [60.5–117]	79.0 [59.0–118]	0.045
Sleep efficiency (%), median [Q1–Q3]	88.0 [80.3–92.7]	90.0 [83.8–93.7]^C,I,O^	88.1 [81.3–92.3]^R,C,O^	85.0 [77.0–91.2]^R,I^	83.3 [76.4–90.0]^R,I,O^	<0.0001
Epworth sleepiness scale (ESS), median [Q1–Q3]	6.00 [3.00–9.00]	5.00 [3.00–8.00]	6.00 [3.00–9.00]	6.00 [4.00–9.00]	6.00 [3.00–9.00]	0.59
ESS > 10, n/N (%)	237/1791 (13.2%)	93/791 (11.8%)	61/380 (16.1%)	56/421 (13.3%)	27/199 (13.6%)	0.25
PSQI score, median [Q1–Q3]	4.00 [3.00–7.00]	4.00 [2.00–5.00]^C,I^	7.00 [5.00–10.0]^R,O^	4.00 [2.00–5.00]^C,I^	7.00 [5.00–10.0]^R,O^	<0.0001

Categorical variables are presented as n/N (%), where N is the number of participants with available data for the corresponding variable. Continuous variables are presented as median [25th–75th percentile] and were calculated using available observations. Variable-specific missing data are reported in Supplementary Table S2. Data were analyzed using Pearson’s chi-squared test, or the Kruskal–Wallis test, as appropriate. Pairwise p-values were corrected for multiple comparisons using the Benjamini-Hochberg method.

BMI: Body Mass Index; CVE: Cardiovascular events; AHI: Apnea-Hypopnea Index; ODI: Oxygen Desaturation Index; PLMSI: Periodic Leg Movement in Sleep Index; PWAD: Pulse Wave Amplitude Drops; T90: percentage of total sleep time with oxygen saturation below 90%; RMSSD: Root Mean Square of Successive Differences; SDNN: Standard Deviation of NN intervals; pNN50: percentage of NN intervals that differ >50 ms from each other; TST: Total Sleep Time; WASO: Wake After Sleep Onset; REM: Rapid Eye Movement; PSQI: Pittsburgh Sleep Quality Index. R: Reference, I: Insomnia, C: COMISA, O: OSA: significant p-value of the pairwise comparison with the corresponding group.

Compared with the reference group, participants in the COMISA group were older, more frequently men, had a higher BMI, and a greater prevalence of cardiovascular comorbidities. There were no significant differences in age, sex, BMI, AHI, or comorbidities between the OSA and COMISA groups. COMISA participants showed a lower proportion of slow wave sleep compared to both the insomnia and OSA groups. Compared with the reference group, participants in the insomnia group were predominantly women and slightly older, although they were younger than participants in the OSA and COMISA groups.

### Main analysis

When moderate-to-severe OSA was analyzed irrespective of insomnia symptoms, it was significantly associated with incident cardiovascular events (crude HR 3.17 [2.27–4.43]; p < 0.0001; adjusted HR 1.73 [1.18–2.56]; p = 0.0060).

We then evaluated COMISA using a single variable with four levels (reference, insomnia alone, OSA without insomnia, COMISA). Kaplan–Meier curves showed significant differences in event-free survival across groups (p < 0.0001; Fig. 2).

**Fig. 2:**
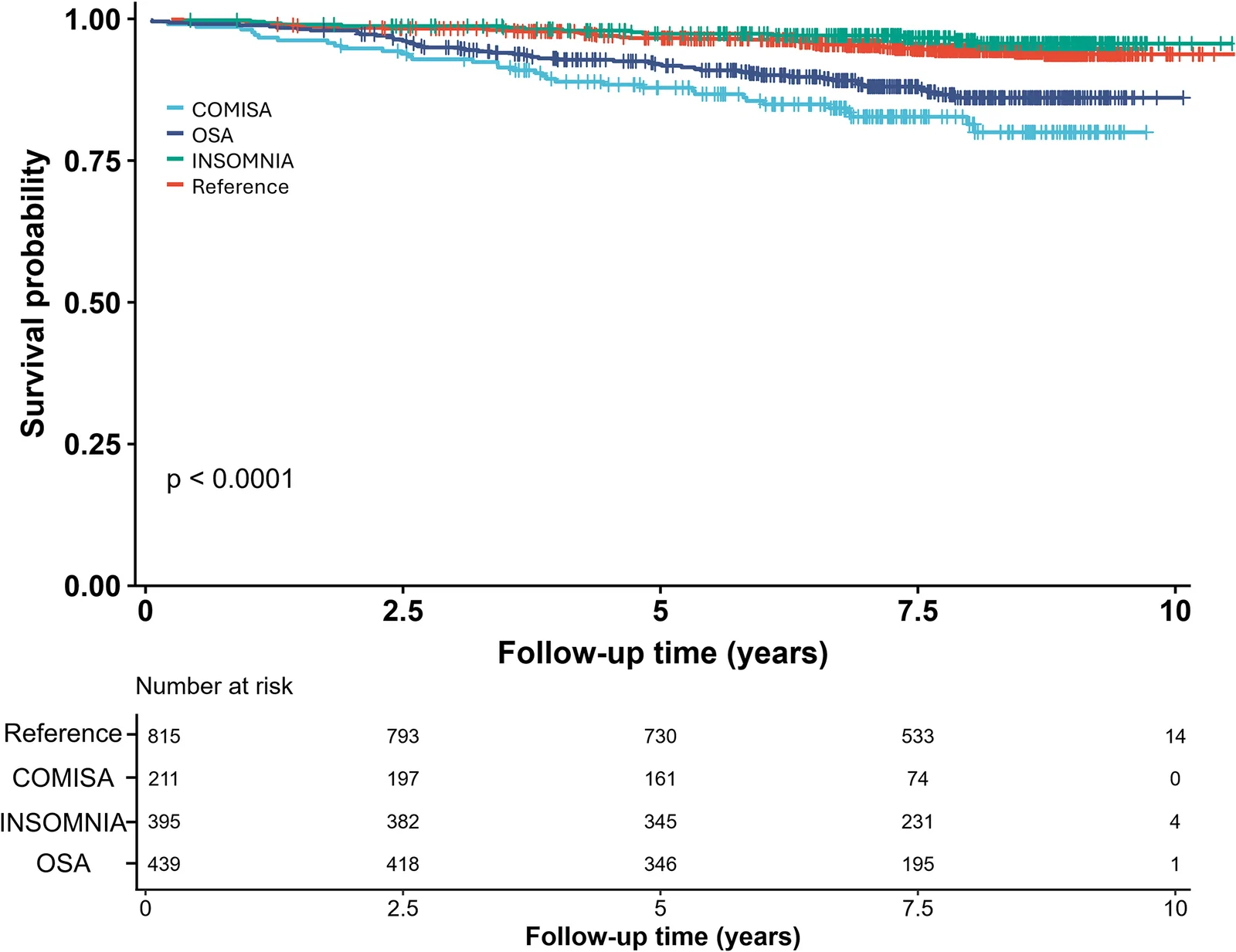
**Kaplan–Meier curves for incident cardiovascular events according to sleep status groups.** Kaplan–Meier survival curves are shown for the four groups: reference (no insomnia, no OSA), insomnia alone, OSA alone, and COMISA. The number of participants at risk at each time point is displayed below the plot. The p-value corresponds to the log-rank test comparing survival curves across groups.

Cox proportional hazards models showed that the COMISA group was significantly associated with a higher risk of incident cardiovascular events compared with the reference group (crude HR 3.68 [2.35–5.76]; p < 0.0001; adjusted HR 1.78 [1.09–2.92]; p = 0.021). The adjusted 8-year absolute risk of cardiovascular events was 12.9% in participants with COMISA compared with 8.1% in the reference group, an absolute difference of 4.7 percentage points. In contrast, neither insomnia alone (adjusted HR 0.59 [0.31–1.11]; p = 0.10) nor OSA without insomnia (adjusted HR 1.33 [0.84–2.10]; p = 0.22) was significantly associated with incident cardiovascular events (Fig. 3A; Supplementary Table S3). The model including an interaction term between OSA and insomnia showed a significant interaction (HR for interaction 2.28 [1.06–4.90]; p = 0.035; Supplementary Table S4).

**Fig. 3:**
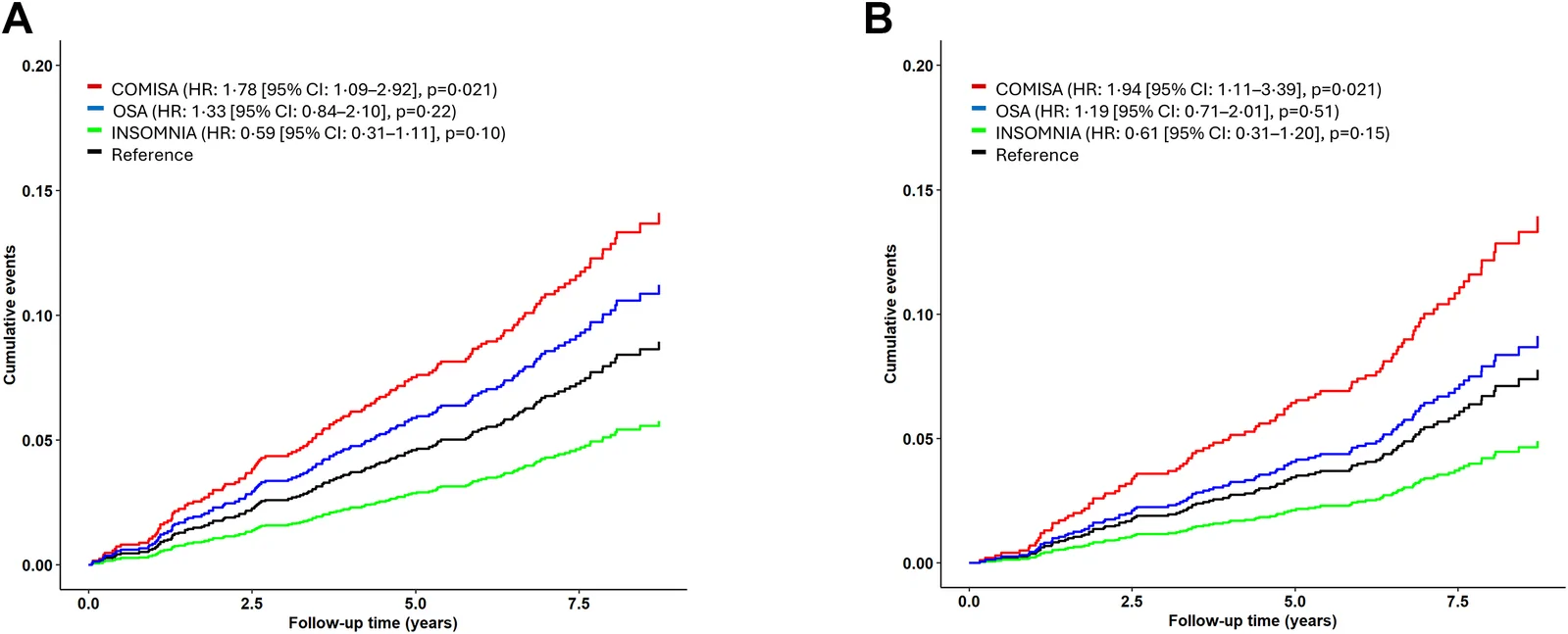
**Adjusted cumulative incidence of cardiovascular events according to sleep disorder group.** (A) Full sample including participants with and without prior cardiovascular events (n = 1845). (B) Participants without prior cardiovascular events (n = 1791). Curves represent model-based cumulative incidence estimates derived from fully adjusted Cox proportional hazards models for four groups: reference (no insomnia, no OSA), insomnia alone, OSA alone, and COMISA. Models were adjusted for age, sex, body mass index category, diabetes, dyslipidemia, hypertension, smoking status, alcohol intake, and previous cardiovascular events (panel A only). Hazard ratios (HRs) with 95% confidence intervals are displayed in the figure.

When analyses were restricted to participants without prior cardiovascular events, COMISA remained significantly associated with incident cardiovascular outcomes (crude HR 3.13 [1.85–5.31], p < 0.0001; adjusted HR 1.94 [1.11–3.39]; p = 0.021), whereas insomnia alone (adjusted HR 0.61 [0.31–1.20]; p = 0.15) and OSA alone (adjusted HR 1.19 [0.71–2.01]; p = 0.51) were not (Fig. 3B; Supplementary Table S3). In this subgroup, the interaction between OSA and insomnia was statistically significant (HR for interaction 2.67 [1.12–6.39]; p = 0.027; Supplementary Table S4).

The association between COMISA and incident cardiovascular events was consistent across alternative insomnia definitions. In the full sample, COMISA remained significantly associated with incident cardiovascular events when defined using subjective prolonged WASO (HR 1.86 [1.12–3.10]; p = 0.017; Fig. 4A), objective prolonged WASO (HR 1.96 [1.19–3.20]; p = 0.0080; Fig. 4B), and regular sleep medication use (HR 2.14 [1.31–3.49]; p = 0.0024; Fig. 4C). Results were similar in analyses restricted to participants without prior cardiovascular events, with significant associations observed using subjective prolonged WASO (adjusted HR 2.34 [1.32–4.14]; p = 0.0034; Fig. 4D), objective prolonged WASO (adjusted HR 2.20 [1.26–3.85]; p = 0.0058; Fig. 4E), and sleep medication use (HR 2.28 [1.32–3.92]; p = 0.0030; Fig. 4F).

**Fig. 4:**
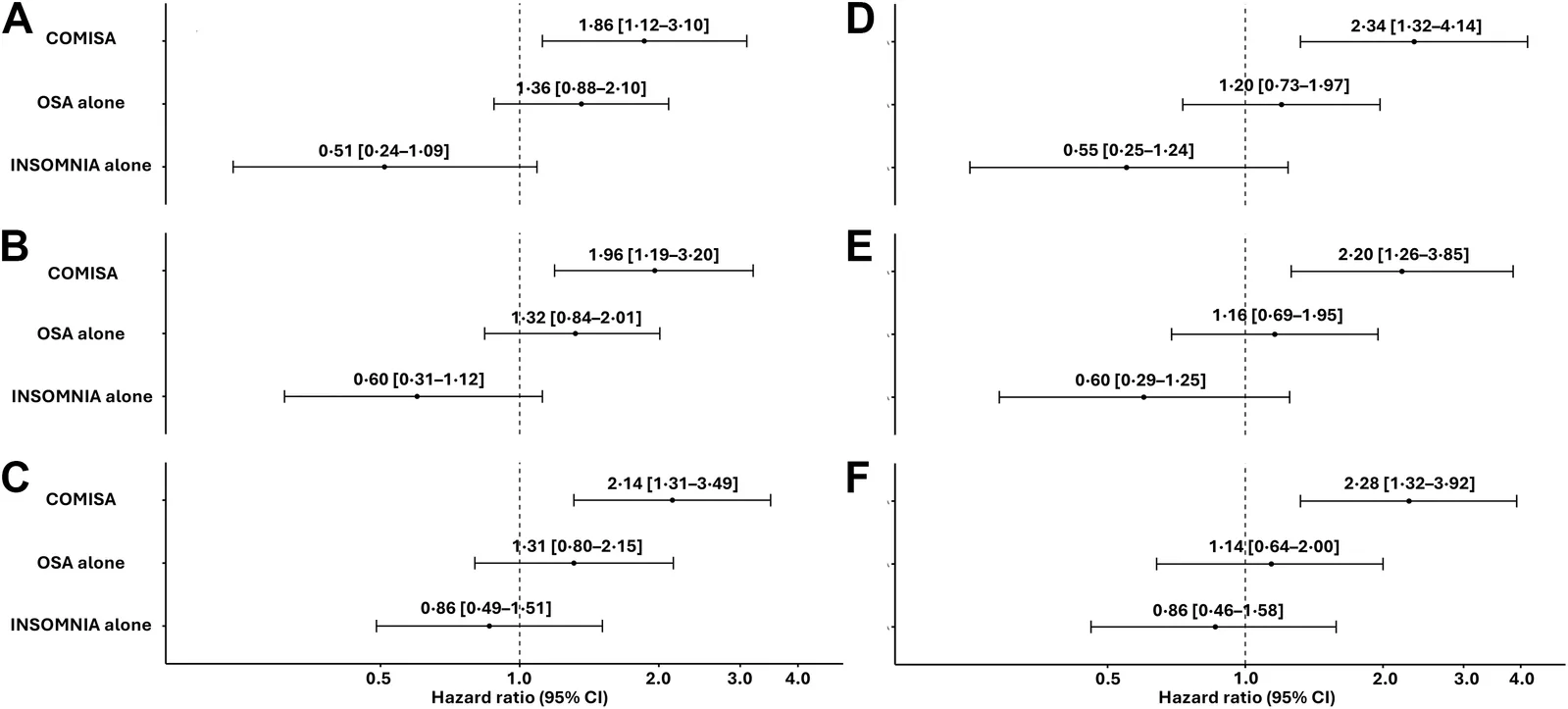
**Sensitivity analyses: adjusted hazard ratios for incident cardiovascular events according to sleep disorder group using alternative definitions of insomnia.** Forest plots display adjusted hazard ratios (HRs) with 95% confidence intervals for insomnia alone, OSA alone, and COMISA. Analyses were restricted to participants for whom all three alternative insomnia definitions could be applied. Panels A–C included 1821 participants with and without prior cardiovascular events and panels D–F included 1768 participants without prior cardiovascular events. Insomnia was defined as follows: (A, D) difficulty falling asleep within 30 min and/or maintaining sleep combined with subjective WASO ≥30 min during the past month (PSQI items 1–4); (B, E) difficulty initiating and/or maintaining sleep combined with objectively measured wake after sleep onset (WASO) ≥30 min on polysomnography; (C, F) difficulty initiating and/or maintaining sleep and/or use of sleep medication ≥3 times per week (PSQI item 7). All models were adjusted for age, sex, body mass index category, smoking status, alcohol intake, hypertension, diabetes, dyslipidemia, and prior cardiovascular events (panels A–C only).

### Sensitivity analyses

After excluding participants who initiated CPAP treatment during follow-up, COMISA remained associated with incident cardiovascular events both in the full sample (adjusted HR 1.89 [1.13–3.16]; p = 0.016; Supplementary Fig. S1A) and among participants without prior cardiovascular events (adjusted HR 1.82 [1.01–3.29]; p = 0.045; Supplementary Fig. S1B).

Including patients with total sleep time <240 min did not alter the associations. COMISA remained associated with incident cardiovascular events both in the full sample (adjusted HR 1.73 [1.06–2.81]; p = 0.029; Supplementary Fig. S2A) and among participants without prior cardiovascular events (adjusted HR 1.89 [1.08–3.29]; p = 0.025; Supplementary Fig. S2B).

After excluding cerebrovascular events from the composite outcome (N = 35), COMISA was no longer significantly associated with incident cardiovascular events either in the full sample (adjusted HR 1.43 [0.79–2.58]; p = 0.23; Supplementary Fig. S3A) or among participants without prior cardiovascular events (adjusted HR 1.43 [0.69–2.94]; p = 0.34; Supplementary Fig. S3B).

## Discussion

In this large, population-based cohort with adjudicated cardiovascular outcomes and more than seven years of follow-up, COMISA was prospectively associated with an increased risk of incident cardiovascular events. This association remained significant after adjustment for established cardiovascular risk factors, including prior cardiovascular events, and was not observed for insomnia or OSA alone. These results support a negative effect of the co-occurrence of insomnia symptoms and OSA on cardiovascular health. A graphical summary of the study design and main findings is provided in Fig. 5.

**Fig. 5:**
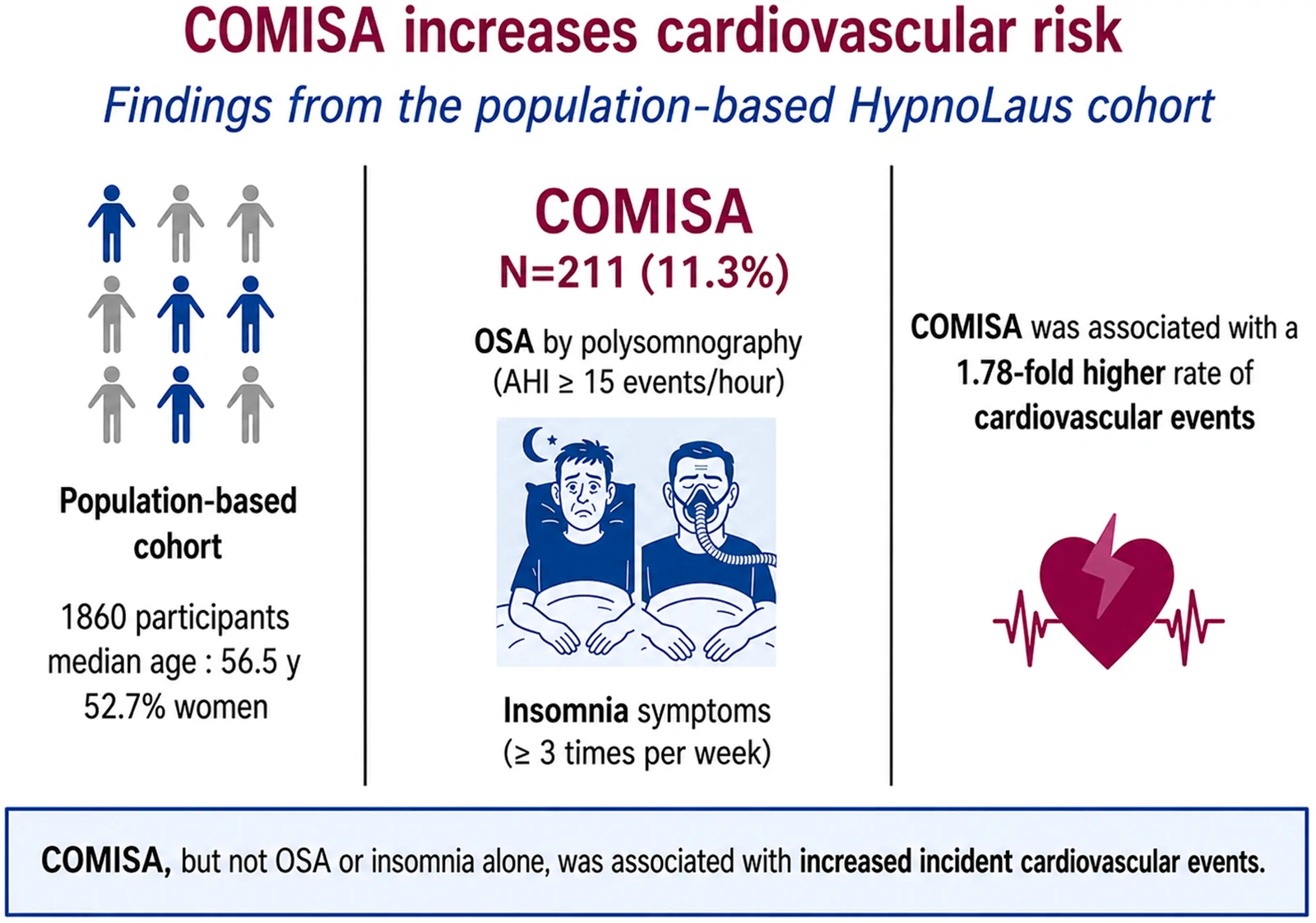
**Graphical summary of the study design and main findings.** Among 1860 participants from the population-based HypnoLaus cohort, 211 (11.3%) had COMISA, defined by the co-occurrence of insomnia symptoms (≥3 times per week) and OSA (AHI ≥15 events/h). COMISA was associated with a higher risk of incident cardiovascular events (adjusted HR 1.78, 95% CI 1.09–2.92), whereas OSA or insomnia alone were not significantly associated with cardiovascular events.

The lower estimate observed for insomnia alone should be interpreted cautiously. It was not statistically significant and was not consistent across model specifications. In additional analyses modeling insomnia and OSA as separate binary variables, insomnia was not associated with incident cardiovascular events, suggesting that this finding likely reflects the specific composition of the insomnia-without-OSA subgroup rather than a true protective effect.

Moderate-to-severe OSA, irrespective of insomnia symptoms, was associated with increased cardiovascular risk in our study, consistent with existing literature. However, when distinguishing patients according to the presence of insomnia, only COMISA remained significantly associated with cardiovascular risk. While insomnia has been linked to modest increases in cardiovascular outcomes in recent meta-analyses,^23^^,^^24^ these studies generally did not account for coexisting OSA. This limitation may contribute to the observed discrepancies and suggests that the excess cardiovascular risk may be primarily driven by the COMISA phenotype rather than by insomnia or OSA alone.

Previous studies, including our own cross-sectional work in three international population-based cohorts, have reported varying prevalence estimates of COMISA, influenced by insomnia definitions, OSA assessment methods, and population characteristics.^6^^,^^25^ Only a few longitudinal studies have examined cardiovascular events in relation to both insomnia complaints and OSA as defined by objectively measured AHI. In a multicenter cohort of elderly patients with objectively diagnosed OSA (AHI ≥15/hour by polysomnography) and insomnia (ICSD-3 criteria), COMISA was associated with a higher risk of major adverse cardiovascular events, particularly unstable angina, with the strongest associations observed in men under 70 years of age who were overweight or obese. In this study, cardiovascular risk was compared between patients with COMISA and those without COMISA, but the independent effects of insomnia or OSA alone were not assessed.^12^ In the Sleep Heart Health Study (SHHS) cohort (N = 4160), individuals with COMISA defined by an AHI ≥15 events/hour and frequent insomnia symptoms (≥16 nights/month) with daytime impairment had a twofold unadjusted risk of incident cardiovascular events over 11 years of follow-up. However, this association was no longer significant after adjustment for established cardiovascular risk factors. Of note, participants with prior cardiovascular events were excluded from the analysis, as in our secondary analysis, whereas participants with heart failure were included in the study outcomes. Notably, a sensitivity analysis using an insomnia definition similar to ours (without daytime impairment) found a significant association with incident cardiovascular events.^11^ Another study based on clinical data from sleep medicine centers (N = 3951; 226 controls, 2107 with OSA alone, 276 with insomnia alone, and 1342 with COMISA) assessed the risk of developing major adverse cardiovascular events using diagnostic codes rather than objective sleep measurements. COMISA was associated with a significantly increased risk of cardiovascular events, particularly among younger adults, although this association was no longer significant after adjustment for comorbidities.^13^ Finally, a large-scale preprint study conducted among U.S. Veterans (N = 937,598; median age 41 years; 12% women) used administrative and electronic health record data to identify insomnia and OSA diagnoses based on ICD-10 (International Statistical Classification of Diseases and Related Health Problems 10th) codes. In this cohort, COMISA was associated with a higher risk of incident hypertension and cardiovascular events compared with OSA or insomnia alone, with adjusted hazard ratios for cardiovascular events reaching 3.81 in men and 3.44 in women.^14^

Together, these studies provide valuable insights into the potential cardiovascular risk associated with COMISA, yet each presents specific limitations. Only two used objective definitions of OSA based on polysomnography. Moreover, two large-scale studies relied solely on electronic health records and diagnostic codes, without objective sleep measurements, which may limit phenotypic precision. In contrast, our study combines full-night polysomnography with detailed symptom-based insomnia assessment in a well-characterized, population-based cohort. This design provides robust evidence that COMISA, but not insomnia or OSA alone, is independently associated with incident cardiovascular events in a middle-aged general population. The adverse synergistic association of COMISA on cardiovascular events may help explain the limited efficacy of continuous positive airway pressure in preventing cardiovascular disease in randomized controlled trials, highlighting the need to consider comorbid insomnia as a potential effect modifier. Our study extends the current literature by providing prospective evidence based on PSG-defined OSA and adjudicated cardiovascular events, using consistent and replicable definitions in a large general population sample.

Interestingly, the higher risk observed when including sleep medication use in the insomnia definition may reflect a subgroup with more persistent or severe insomnia, as pharmacological treatment can partially mask symptoms without addressing the underlying disorder. In addition, the use of hypnotic medications, particularly benzodiazepines, has been associated with increased cardiovascular risk,^26^ suggesting that both insomnia severity and treatment-related factors may contribute to this finding.

The statistical interaction between OSA and insomnia indicates that the co-occurrence of both conditions may confer a higher cardiovascular risk than either condition alone. Several pathophysiological mechanisms may explain the increased cardiovascular risk in COMISA. Insomnia can contribute to hyperarousal, dysregulation of the hypothalamic-pituitary-adrenal axis, increased sympathetic tone, and metabolic disturbances.^9^ OSA adds repetitive hypoxia, oxidative stress, intrathoracic pressure swings, inflammation, and further sympathetic activation.^8^^,^^27^ Conventional markers of OSA severity, including AHI, oxygen desaturation index, the percentage of total sleep time spent with oxygen saturation below 90% in total sleep time, hypoxic burden, or the respiratory arousal index, were not significantly different when directly comparing COMISA and OSA in our study. Other cardiovascular risk biomarkers such as pulse wave amplitude drops (PWAD)^28^ and heart rate response to respiratory events (ΔHR)^29^^,^^30^ were previously linked to adverse cardiovascular outcomes in OSA. They may help to better differentiate these populations. However, no COMISA-specific differences were observed either for those markers in our bivariate analysis. Beyond respiratory indices, COMISA was characterized in our cohort by a lower proportion of N3 sleep compared to OSA alone, reflecting altered sleep macrostructure. In the HypnoLaus study, reduced delta spectral power was previously associated with hypertension,^31^ suggesting that microstructural alterations of slow-wave sleep may also be relevant. Together, these findings support the hypothesis that, while respiratory severity does not differ, alterations in both macrostructure (including total sleep time) and microstructure of sleep could be key determinants of the increased cardiovascular risk in COMISA and warrant further investigation. Advanced EEG microstructure analyses, similar to those already applied in insomnia research,^32^ may help disentangle these mechanisms and identify specific electrophysiological signatures of vulnerability in COMISA. A recent heart rate variability (HRV)-based study in the SHHS cohort (N = 5335) reported COMISA-specific autonomic dysfunction, with reduced parasympathetic activity during wakefulness and increased sympathetic activation during sleep, supporting its distinct physiological impact on cardiovascular risk.^33^ In our cohort, baseline heart rate was similar across groups, and some of the main time-domain HRV indices (RMSSD, pNN50) did not differ significantly, apart from a modest variation in SDNN without a consistent COMISA-specific pattern. In the SHHS, differences in HRV markers were accompanied by significant variations in baseline heart rate between groups, which could partly influence autonomic comparisons. The co-occurrence of insomnia and OSA may still amplify these mechanisms in a synergistic manner, but the magnitude of autonomic alterations might depend on underlying cardiovascular and autonomic profiles in each population.

The assessment of insomnia symptoms in our study has certain limitations, particularly regarding the potential misclassification of WASO, which may reflect brief respiratory-related arousals rather than sustained awakenings indicative of genuine difficulties returning to sleep. To address this, sensitivity analyses were performed using both subjective and objective definitions of prolonged WASO (≥30 min). In both cases, the association between COMISA and incident cardiovascular events was of greater magnitude compared to the main analysis. These findings suggest that OSA with prolonged awakenings reflects true difficulty returning to sleep and may carry a higher cardiovascular risk than OSA with brief arousals, which may not represent clinically meaningful insomnia. Endotypic differences of OSA may also contribute to the increased vulnerability observed in COMISA. A prior study has reported an overrepresentation of the low arousal threshold OSA phenotype in this population, potentially leading to greater sleep fragmentation and autonomic instability.^34^ These endotypes may differ across populations, underscoring the need for future research to better characterize their prevalence and impact.

Finally, in our prospective cohort, sensitivity analyses excluding cerebrovascular events from the composite cardiovascular endpoint showed a convergence of COMISA survival with that of OSA alone. Interpretation is limited by the small number and heterogeneity of cerebrovascular events (ischemic and hemorrhagic strokes and central retinal artery occlusion); however, given established links between OSA and stroke,^35^^,^^36^ between insomnia and stroke,^37^^,^^38^ and the prevalence of COMISA,^6^ stroke-focused studies should systematically ascertain and adjust for insomnia symptoms in OSA and conversely consider OSA when evaluating insomnia.

These results emphasize the importance of systematically screening for and managing both insomnia and OSA in clinical settings. Patients with COMISA may represent a particularly vulnerable phenotype with increased cardiovascular risk. Addressing insomnia through cognitive behavioral therapy (CBT-I) has been shown to improve CPAP adherence and may also reduce cardiovascular risk.^39^, ^40^, ^41^ Furthermore, treating OSA may alleviate insomnia symptoms in a subset of patients, suggesting potential bidirectional benefits of integrated treatment approaches.

The main strengths of this study include the large, well-characterized, population-based cohort; objective assessment of OSA using full-night polysomnography; a longitudinal follow-up exceeding seven years with expert-adjudicated cardiovascular events; and multivariable adjustment. The consistency of findings across multiple COMISA definitions and their replication in analyses restricted to participants without prior cardiovascular events further support the robustness of the conclusions.

Nevertheless, several limitations must be acknowledged. Insomnia was assessed using the PSQI, which does not fully align with ICSD-3 or DSM-5 diagnostic criteria. Therefore, we cannot refer to chronic insomnia, and our findings should not be generalized to patients with chronic insomnia. Because insomnia symptoms were assessed at baseline only, they may have changed during follow-up. Our results should therefore be interpreted as reflecting the prognostic value of baseline COMISA rather than persistent insomnia over time. In addition, although we adjusted for a broad range of potential confounders, residual confounding cannot be entirely excluded.

In conclusion, COMISA was independently associated with an increased risk of incident cardiovascular events in this large, population-based cohort, even after accounting for traditional cardiovascular risk factors and pre-existing cardiovascular events. These findings highlight the clinical relevance of identifying and treating COMISA as a distinct and high-risk phenotype. Future research should aim to refine phenotyping through sleep endotyping and physiological biomarkers, and to assess the impact of combined insomnia and OSA treatment strategies on cardiovascular outcomes.

## Contributors

GS: Conceptualization, Data curation, Formal analysis, Investigation, Methodology, Supervision, Writing—original draft, Writing—review and editing.

TI: Conceptualization, Data curation, Formal analysis, Investigation, Methodology, Writing—original draft, Writing—review and editing.

RH: Data curation, Investigation, Methodology, Project administration, Supervision, Funding acquisition, Resources, Writing—review and editing.

NAM: Writing—review and editing.

SB: Writing—review and editing.

SVDB: Writing—review and editing.

GC: Writing—review and editing.

GH: Writing—review and editing.

AW: Writing—review and editing.

IE: Writing—review and editing.

VB: Writing—review and editing.

ASL: Writing—review and editing.

JHR: Writing—review and editing.

PV: Writing—review and editing.

PMV: Writing—review and editing.

GS and TI directly accessed and verified the underlying data. GS and RH were responsible for the decision to submit the manuscript. All authors read and approved the final version of the manuscript.

## Data sharing statement

Coded individual-level data from the CoLaus|PsyCoLaus study are available upon reasonable request to researchers who meet the data-sharing criteria of the CoLaus|PsyCoLaus Datacenter (CHUV, Lausanne, Switzerland). Access is subject to approval by the Scientific Committee and compliance with applicable Swiss data-protection requirements. Any researcher affiliated to a public or private research institution who complies with the CoLaus|PsyCoLaus standards can submit a research application to research.colaus@chuv.ch or research.psycolaus@chuv.ch. Proposals will be evaluated by the Scientific Committee (SC) of the CoLaus|PsyCoLaus study. Detailed instructions for gaining access to the CoLaus|PsyCoLaus data used in this study are available at www.colaus-psycolaus.ch/professionals/how-to-collaborate/.

## Declaration of interests

GS declares unrestricted grants from the Ligue pulmonaire vaudoise and Idorsia, consulting fees from Wellnest Retreats, honoraria for lectures or educational events from Idorsia and advisory board fees from Idorsia; all payments were made to his institution.

PMV declares unrestricted grant from Swiss National Science Foundation; travel support from the European Society of Cardiology; advisory board fees from Santé Publique France; financial interests from La Caixa Foundation as a reviewer of research funding applications.

RH declares unrestricted grants from the Ligue pulmonaire vaudoise, Idorsia, and the Placide Nicod Foundation; consulting fees from Apnimed, SleepRes, and BMC; honoraria for lectures or educational events from Inspire, Nestlé, Löwenstein Medical, ResMed, Bioprojet, Philips, and Medtronic; travel support from ResMed; and advisory board fees from Nyxoah; all payments were made to his institution.

SB declares personal consulting fees from Air Liquide International and ResMed, personal honoraria from ResMed, VitalAire, and Bioprojet, and support for attending meetings or travel from VitalAire and Bioprojet.

ASL declares honoraria for lectures or educational events from Idorsia.

All remaining co-authors declare no competing interests.
