# Supplementary material for: Comorbid insomnia and sleep apnea (COMISA) and incident cardiovascular risk: a prospective analysis from the HypnoLaus cohort
Source: eClinicalMedicine. 2026 Sep 28;100:104227. doi: 10.1016/j.eclinm.2026.104227 (PMC13651482; doi:10.1016/j.eclinm.2026.104227)
Supplement: Supplementary file 1 [file mmc1.pdf]

Comorbid Insomnia and Sleep Apnea (COMISA) and Incident Cardiovascular Risk: A Prospective Analysis from the HypnoLaus Cohort

Geoffroy Solelhac, Théo Imler, Nicola-Andrea Marchi, Sebastien Baillieul, Sandra Van Den Broecke, Gabriela Caetano, Gregory Heiniger, Adrien Waeber, Isabel Ericson, Virginie Bayon, Anne-Sophie Lombardi, Jose Haba-Rubio, Peter Vollenweider, Pedro Marques-Vidal, Raphaël Heinzer

Supplementary Appendix

Table of contents

TABLE S1. DISTRIBUTION OF INCIDENT CARDIOVASCULAR EVENTS IN THE FULL SAMPLE AND IN PARTICIPANTS WITHOUT PRIOR CARDIOVASCULAR EVENTS..2

TABLE S2. MISSING DATA BY VARIABLE...3

TABLE S3. UNADJUSTED AND ADJUSTED COX PROPORTIONAL HAZARDS MODELS FOR INCIDENT CARDIOVASCULAR EVENTS IN THE FULL SAMPLE AND IN PARTICIPANTS WITHOUT PRIOR CARDIOVASCULAR EVENTS 4

TABLE S4. UNADJUSTED AND ADJUSTED COX PROPORTIONAL HAZARDS MODELS FOR INCIDENT CARDIOVASCULAR EVENTS IN THE FULL SAMPLE AND IN PARTICIPANTS WITHOUT PRIOR CARDIOVASCULAR EVENTS WITH INTERACTION. 5

FIGURE S1. ADJUSTED CUMULATIVE INCIDENCE OF CARDIOVASCULAR EVENTS AFTER EXCLUSION OF PARTICIPANTS WHO INITIATED CPAP TREATMENT DURING FOLLOW-UP 6

FIGURE S2. ADJUSTED CUMULATIVE INCIDENCE OF CARDIOVASCULAR EVENTS WITHOUT EXCLUSION OF PARTICIPANTS WITH SHORT SLEEP DURATION (<4 HOURS)................................7

FIGURE S3. ADJUSTED CUMULATIVE INCIDENCE OF CARDIOVASCULAR EVENTS AFTER EXCLUSION OF CEREBROVASCULAR EVENTS FROM THE OUTCOME 8

	With and without previous CVE<br>N=143	Without previous CVE<br>N=102
CHD, n (%)	55 (38%)	32 (31%)
AMI, n (%)	41 (29%)	34 (33%)
Death from CVD, n (%)	12 (8%)	8 (8%)
Ischemic stroke, n (%)	24 (17%)	19 (19%)
Hemorrhagic stroke, n (%)	10 (7%)	8 (8%)
Central retinal artery occlusion, n (%)	1 (1%)	1 (1%)

Table S1. Distribution of incident cardiovascular events in the full sample and in participants without prior cardiovascular events.
Events are presented for the entire sample (with and without prior cardiovascular events) and for the subgroup of participants free of prior cardiovascular events at baseline.
AMI: acute myocardial infarction, CHD: coronary heart disease, CVD: cardiovascular disease, CVE: cardiovascular events.

Variable	Missing n/N (%)
BMI (continuous, kg/m^2)	11/1860 (<1%)
Hypertension (categorical)	2/1860 (<1%)
Diabetes (categorical)	2/1860 (<1%)
AHI (continuous, events/h)	0/1860 (0%)
Previous history of CVE (categorical)	0/1860 (0%)
Age (continuous, years)	0/1860 (0%)
Gender (categorical)	0/1860 (0%)
Current smokers (categorical)	0/1860 (0%)
Alcohol consumption (continuous, unit/week)	0/1860 (0%)
Dyslipidemia (categorical)	2/1860 (<1%)
PSQI sleep time (continuous, hours)	5/1860 (<1%)
PSQI time to fall asleep (continuous, min)	28/1860 (2%)
PSQI wake up time (continuous, hours)	6/1860 (<1%)
PSQI sleep duration (continuous, hours)	12/1860 (1%)
Epworth sleepiness scale (continuous)	69/1860 (4%)

Table S2. Missing data among the 1860 participants included in the primary analytic sample.
BMI: body mass index, AHI: apnea-hypopnea index, CVE: cardiovascular event, PSQI: Pittsburgh sleep quality index

	Full sample				Without prior CVE			
	**Unadjusted model N=1860**		**Adjusted model N=1845**		**Unadjusted model N=1805**		**Adjusted model N=1791**	
	HR [95% CI]	p-value	HR [95% CI]	p-value	HR [95% CI]	p-value	HR [95% CI]	p-value
COMISA	3·68 [2·35–5·76]	<0·0001	1·78 [1·09–2·92]	0·021	3·13 [1·85–5·31]	<0·0001	1·94 [1·11–3·39]	0·021
Insomnia alone	0·69 [0·38–1·26]	0·23	0·59 [0·31–1·11]	0·10	0·73 [0·38–1·40]	0·35	0·61 [0·31–1·20]	0·15
OSA alone	2·47 [1·65–3·71]	<0·0001	1·33 [0·84–2·10]	0·22	1·97 [1·22–3·18]	0·0057	1·19 [0·71–2·01]	0·51
Previous history of CVE	–	–	9·87 [6·35–15·32]	<0·0001	–	–	–	–
Male	–	–	1·21 [0·82–1·80]	0·43	–	–	1·46 [0·93–2·29]	0·10
Age (years)	–	–	1·05 [1·02–1·07]	<0·0001	–	–	1·07 [1·05–1·10]	<0·0001
Diabetes	–	–	1·97 [1·30–2·97]	0·0013	–	–	1·66 [0·97–2·85]	0·066
Hypertension	–	–	1·57 [1·01–2·44]	0·046	–	–	1·44 [0·91–2·28]	0·12
Current smoker	–	–	2·35 [1·56–3·54]	<0·0001	–	–	2·54 [1·63–3·96]	<0·0001
Alcohol consumption (unit/week)	–	–	0·99 [0·97–1·02]	0·65	–	–	0·97 [0·93–1·00]	0·041
Dyslipidemia	–		1·27 [0·87–1·87]	0·22	–		1·18 [0·75–1·86]	0·47
BMI category	–	–			–	–		
Normal, <25 kg/m^2	–	–	–	–	–	–	–	–
Overweight, ≥25 and <30 kg/m^2	–	–	1·03 [0·68–1·58]	0·88	–	–	1·00 [0·62–1·62]	0·99
Obese, ≥30 kg/m^2	–	–	0·81 [0·46–1·39]	0·43	–	–	0·91 [0·47–1·76]	0·78

Table S3. Unadjusted and adjusted Cox proportional hazards models for incident cardiovascular events in the full sample and in participants without prior cardiovascular events.
BMI: body mass index, CVE: cardiovascular event, HR: Hazard Ratio, PSQI: Pittsburgh sleep quality index

	Full sample				Without prior CVE			
	Unadjusted model N=1860		Adjusted model N=1845		Unadjusted model N=1805		Adjusted model N=1791	
	HR [95% CI]	p-value	HR [95% CI]	p-value	HR [95% CI]	p-value	HR [95% CI]	p-value
OSA main effect	2·47 [1·65–3·71]	<0·0001	1·33 [0·84–2·10]	0·22	1·97 [1·22–3·18]	0·0057	1·19 [0·71–2·01]	0·51
Insomnia main effect	0·69 [0·38–1·26]	0·23	0·59 [0·31–1·11]	0·10	0·73 [0·38–1·40]	0·34	0·61 [0·31–1·20]	0·15
OSA x Insomnia interaction	2·16 [1·03–4·54]	0·041	2·28 [1·06–4·90]	0·035	2·18 [0·94–5·10]	0·071	2·67 [1·12–6·39]	0·027

Table S4. Unadjusted and adjusted Cox proportional hazards models for incident cardiovascular events in the full sample and in participants without prior cardiovascular events with interaction.

In this interaction model, the main effects for OSA and insomnia represent OSA alone versus the reference group and insomnia alone versus the reference group, respectively.

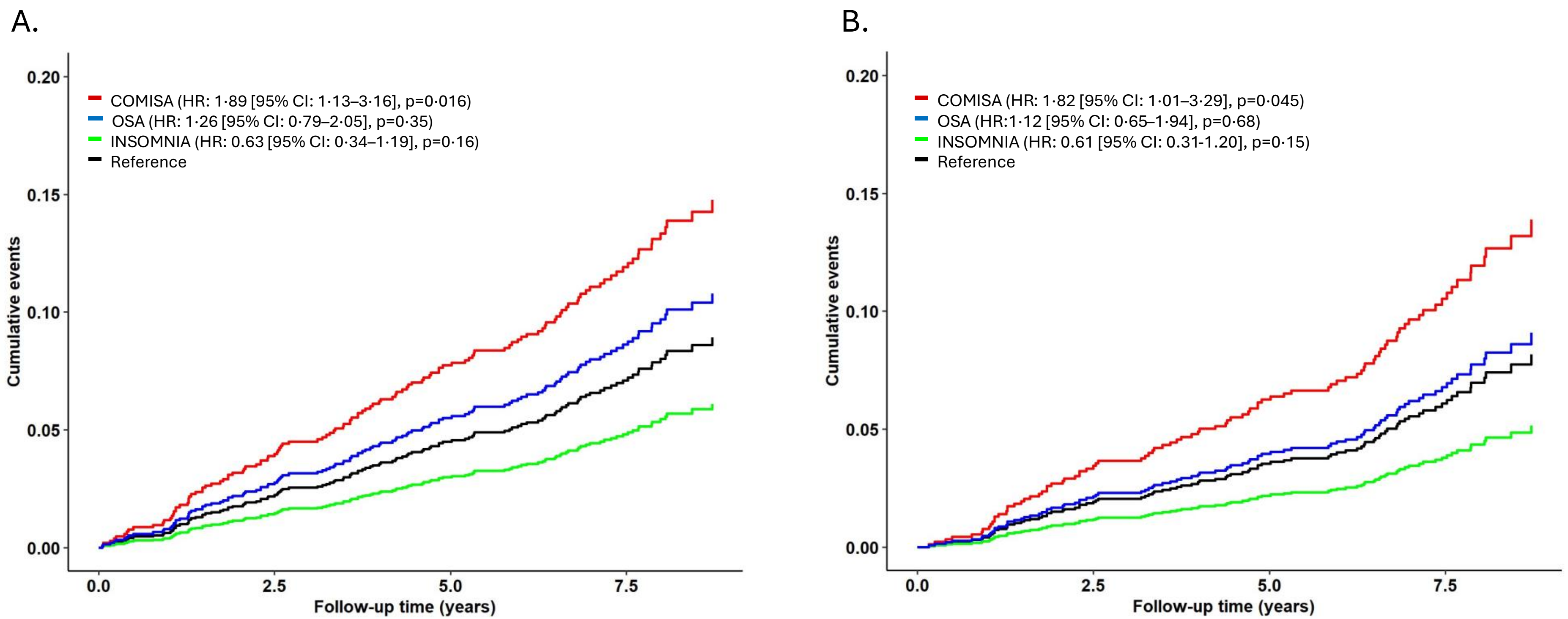

Figure S1. Adjusted cumulative incidence of cardiovascular events after exclusion of participants who initiated CPAP treatment during follow-up.
(A) Full sample including participants with and without prior cardiovascular events (n=1737). (B) Participants without prior cardiovascular events (n=1692). Curves represent model-based cumulative incidence estimates derived from fully adjusted Cox proportional hazards models for the four groups: reference (no insomnia, no OSA), insomnia alone, OSA alone, and COMISA. Models were adjusted for age, sex, body mass index category, diabetes, dyslipidemia, hypertension, smoking status, alcohol intake, and previous cardiovascular events (panel A only). Hazard ratios (HRs) with 95% confidence intervals are displayed in the figure.

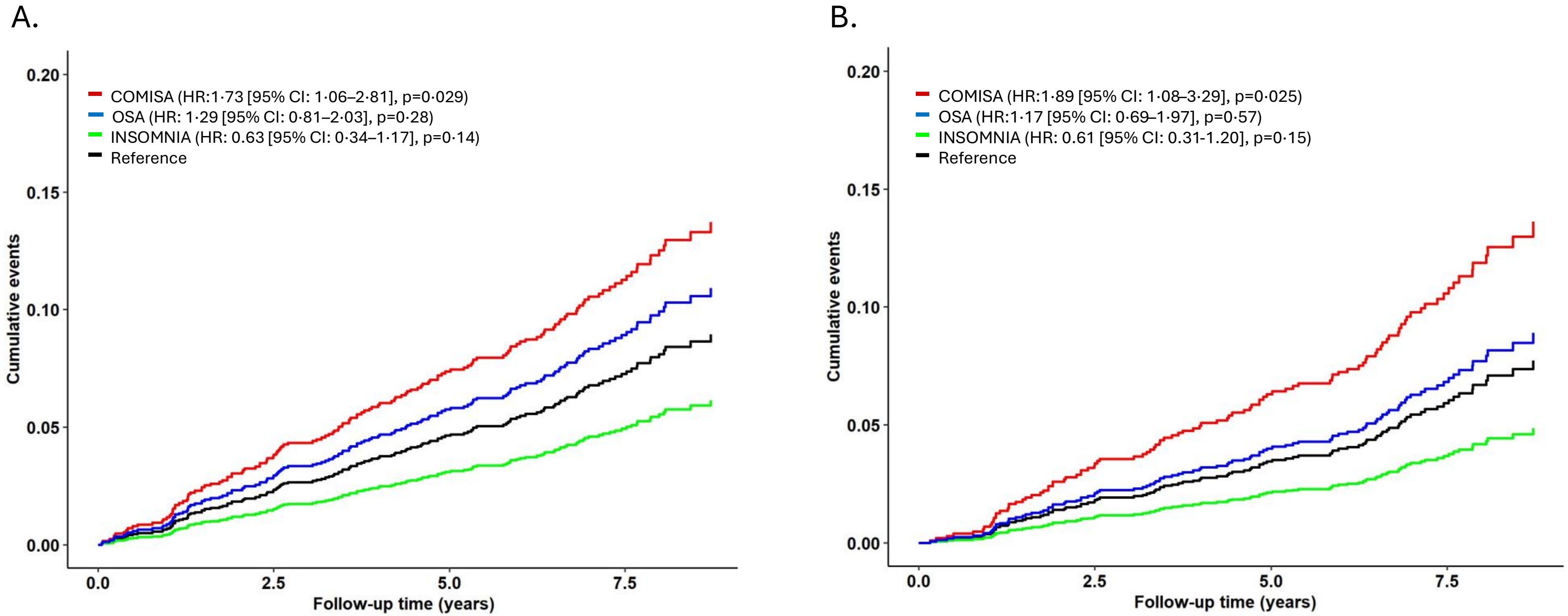

Figure S2. Adjusted cumulative incidence of cardiovascular events without exclusion of participants with short sleep duration (<4 hours).
(A) Full sample including participants with and without prior cardiovascular events (n=1880). (B) Participants without prior cardiovascular events (n=1825). Curves represent model-based cumulative incidence estimates derived from fully adjusted Cox proportional hazards models for the four groups: reference (no insomnia, no OSA), insomnia alone, OSA alone, and COMISA. In contrast to the main analysis, participants with total sleep time <4 hours were not excluded. Models were adjusted for age, sex, body mass index category, diabetes, dyslipidemia, hypertension, smoking status, alcohol intake, and previous cardiovascular events (panel A only). Hazard ratios (HRs) with 95% confidence intervals are displayed in the figure.

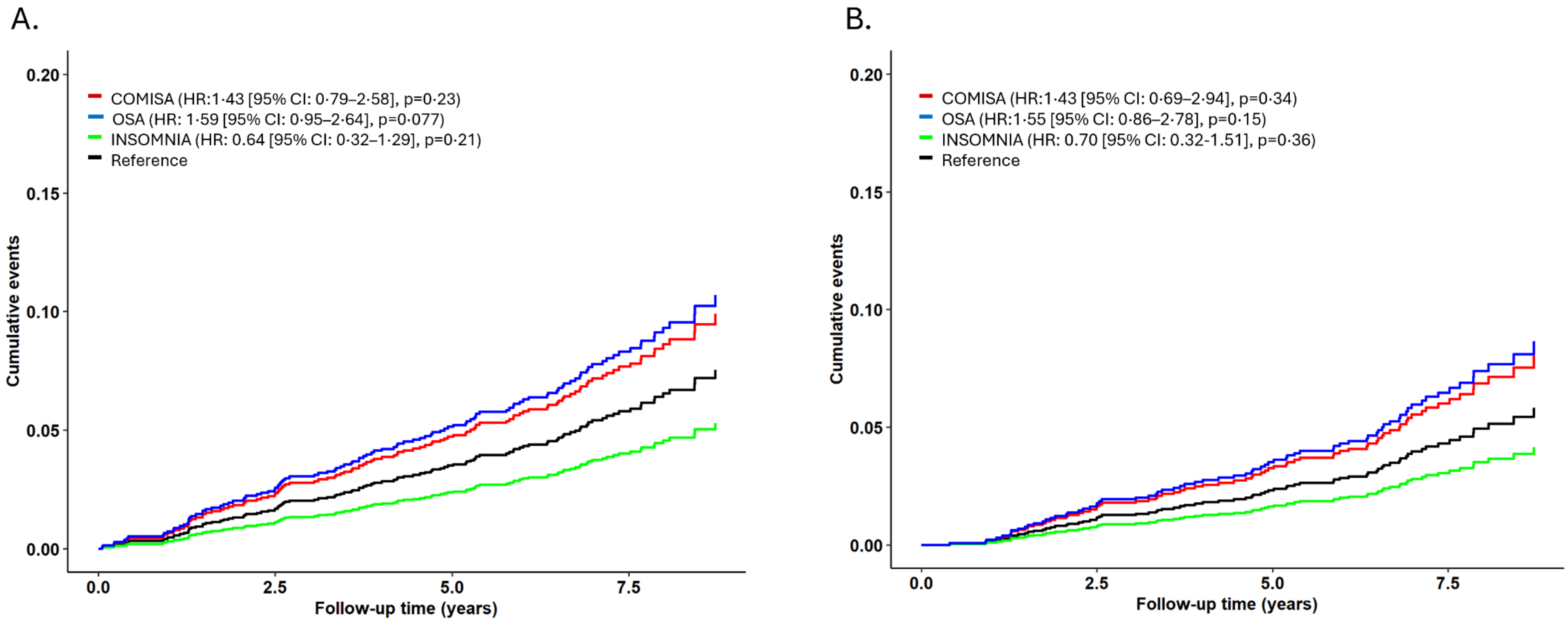

Figure S3. Adjusted cumulative incidence of cardiovascular events after exclusion of 35 cerebrovascular events from the outcome.
(A) Full sample including participants with and without prior cardiovascular events (n=1840). (B) Participants without prior cardiovascular events (n=1789). Curves represent model-based cumulative incidence estimates derived from fully adjusted Cox proportional hazards models for the four groups: reference (no insomnia, no OSA), insomnia alone, OSA alone, and COMISA. The outcome was restricted to cardiac events only (i.e., excluding cerebrovascular events). Models were adjusted for age, sex, body mass index category, diabetes, dyslipidemia, hypertension, smoking status, alcohol intake, and previous cardiovascular events (panel A only). Hazard ratios (HRs) with 95% confidence intervals are displayed in the figure.
